# Comparison of infinitesimal and finite locus models for long-term breeding simulations with direct and maternal effects at the example of honeybees

**DOI:** 10.1371/journal.pone.0213270

**Published:** 2019-03-06

**Authors:** Manuel Plate, Richard Bernstein, Andreas Hoppe, Kaspar Bienefeld

**Affiliations:** Institute for Bee Research Hohen Neuendorf, Hohen Neuendorf, Germany; University of North Carolina at Greensboro, UNITED STATES

## Abstract

Stochastic simulation studies of animal breeding have mostly relied on either the infinitesimal genetic model or finite polygenic models. In this study, we investigated the long-term effects of the chosen model on honeybee breeding schemes. We implemented the infinitesimal model, as well as finite locus models, with 200 and 400 gene loci and simulated populations of 300 and 1000 colonies per year over the course of 100 years. The selection was of a directly and maternally influenced trait with maternal heritability of $h_{m}^{2}=0.42$, direct heritability of $h_{d}^{2}=0.27$, and a negative correlation between the effects of *r_md_* = − 0.18. Another set of simulations was run with parameters $h_{m}^{2}=0.53$, $h_{d}^{2}=0.34$, and *r_md_* = − 0.53. All models showed similar behavior for the first 20 years. Throughout the study, we observed a higher genetic gain in the direct than in the maternal effects and a smaller gain with a stronger negative covariance. In the long-term, however, only the infinitesimal model predicted sustainable linear genetic progress, while the finite locus models showed sublinear behavior and, after 100 years, only reached between 58% and 62% of the mean breeding values in the infinitesimal model. While the infinitesimal model suggested a reduction of genetic variance by 33% to 49% after 100 years, the finite locus models saw a more drastic loss of 76% to 92%. When designing sustainable breeding strategies, one should, therefore, not blindly trust the infinitesimal model as the predictions may be overly optimistic. Instead, the more conservative choice of the finite locus model should be favored.

## Introduction

A major concern in sustainable breeding and conservation programs is the preservation of genetic variance in the population [1–4]. To estimate the development of genetic variance under various conditions, Monte Carlo simulations have been widely applied in animal breeding and conservation genetics since computers were introduced [5, 6], and they remain a valuable tool [7, 8]. Currently, two main types of genetic models are used to investigate developments in genetic variance via simulations: Fisher’s infinitesimal model [9, 10] and the finite locus models [11, 12]. The infinitesimal model assumes that quantitative traits are genetically influenced by an infinitely large number of loci, each of which has the same infinitesimally small impact. In contrast, finite locus models assume a possibly large but finite number of loci contribute to a trait and allow for different magnitudes of influence of the respective loci. Both the infinitesimal model [13, 14] and finite locus models [15, 16] have been used in recent simulation studies.

Although existing software, such as ADAM [17], can run simulations based on either model, comparisons of the two models’ properties in stochastic simulations appears to be scarce in the literature. Fournet-Hanocq & Elsen [18] compared Monte Carlo simulations based on the finite locus model with two deterministic simulations, one of which relied on the infinitesimal model, and found greater losses of genetic variance in the finite locus models. Further studies focused on only one of the models and found dependencies of the simulation outcomes on the population size [19] or the distribution of QTL effects [12]. In [20], simulations using both models were performed. However, this study compared the accuracy of different methods for breeding value estimation depending on the models rather than the properties of the models themselves. We are not aware of any direct comparison of Monte Carlo simulations based on the respective models.

Estimates of the long-term effects of animal breeding, especially the limits of selection possibilities, were introduced in [21], relating the possible total genetic gain to the effective population size. The authors of [19] studied the long-term behavior of Monte Carlo simulations that relied on the infinitesimal model. They found the problem of decreasing genetic variance in very small populations and expressed interest in similar studies with different genetic models. A study of long-term breeding effects involving a finite locus model was conducted in [22]. Here, the authors discovered the possibility of improving long-term responses by enhancing the selection of favorable minor alleles. Breeding schemes can select for either single traits or several intercorrelated traits. Often, these traits are not only determined by single individuals alone but also by their contemporaries via indirect effects [23, 24]. The most prevalent of these indirect effects are probably the so-called maternal effects in which the genetic properties of a dam influence the performance of her offspring [25–27].

Simulation studies have already examined the effects of selective breeding for multiple traits and found that breeding value estimation should consider the correlation between different traits [28, 29]. Traditionally, most of these investigations have been solely based on the infinitesimal model [28, 30, 31]. Most modern finite model-based single trait simulation studies assume that there is a favorable allele and suppose that the allele’s effects follow a heavy-tailed distribution, often realized by a gamma distribution. These assumptions cannot be easily transferred to a multivariate setting. Nevertheless, a number of multi-trait studies with finite locus models exist [29, 32, 33]. However, no common standard methodology has been established so far [34].

Simulation studies that include indirect effects appear to be almost exclusively based on the infinitesimal model [35–39]. It has been found that indirect effects have to be correctly addressed in the breeding value estimation to obtain optimal results and that a negative correlation between direct and indirect effects can severely hamper genetic progress [35, 36]. Moreover, a connection between the presence of indirect genetic effects and increased rates of inbreeding has been drawn [38]. In studies investigating the potential of genomic selection in honeybees, finite locus simulations that include maternal effects were implemented by Gupta, et al. [40, 41].

Strategies used to address the problem of uncertain paternity in several breeding schemes were first developed in [42] and [43]. By implementing computer simulations based on the infinitesimal model, Sullivan [44] showed a clear negative effect of uncertain paternity on breeding success. Cardoso & Tempelman [45] carried out simulation studies with uncertain paternity, including directly and maternally affected traits, and showed that they are equally affected by missing paternal information. Recently, a study conducted by Tonussi et al. [46] investigated the influences of unknown sires on genomic breeding value estimation based on a finite locus model. This study also revealed further implications of unknown paternity in the traditional BLUP setting, including the overestimation of genetic parameters.

The biology and current breeding schemes of honeybee show the aforementioned peculiarities to a heightened degree. They combine negatively correlated maternal (queen) and direct (worker) effects with an uncertain paternal descent and a strong need for the maintenance of genetic diversity, which is threatened by inbreeding depression [47, 48]. Commercial traits, such as honey production or *Varroa* resistance, can be influenced maternally by the queen through her egg laying frequency or pheromone release, as well as directly by the workers (e.g., via nectar collection or hygienic behavior). Maternal and direct effects in honeybees differ slightly from their respective counterparts in mammals in two aspects.

- The direct effect is not attributed to an individual but to the collective of workers in a colony. It is, thus, often seen as their average effect [49].
- The maternal effect of a queen is not directed at the next generation of queens but at her worker group, an entity that is not directly involved in the selection process. Hence, selection for maternal effects does not suffer from the inaccessibility of information in the latest generation, which leads to reduced selection potential in maternally affected traits in other species [50].

Nevertheless, the notion of maternal and direct effects in honeybees resembles corresponding concepts in other farm animals very closely and are regularly seen as their equivalents [49, 51, 52]. This makes the honeybee an ideal model species to quantify the influence of previously unexamined factors.

Moreover, there is a specific current reason to investigate bee breeding. Because of a lack of selection, small honeybee races in Europe get increasingly replaced by two selected subspecies [53]. New breeding programs will soon be set up for these endangered races, and new performance testing protocols are being developed [54–57]. Therefore, at this point, sustainable long-term breeding strategies are needed.

After the establishment of a BLUP-based breeding value estimation for the honeybee [52, 58], a new species-specific methodology was developed [49, 59]. Primary simulation studies have been carried out using either the infinitesimal model [49] or finite locus models [40, 41]. In this work, we will explain the methodological concept, which is also transferrable to other species, and establish simulation procedures for honeybee breeding schemes. We compare the long-term behavior of Monte Carlo simulations based on infinitesimal and finite locus models to facilitate reliable model choices for upcoming population-specific studies.

## Methods

### General assumptions

Honeybee colonies were modelled as consisting of a single queen and her offspring (a group of non-reproducing worker bees). In addition to the diploid queens and workers, haploid drones were individually modelled. After being created, young queens immediately mated with 12 drones each. Afterward, the genetic information of the queen and the 12 drones was used to create a worker group and daughter queens. Drones received their genetic information from their respective dam queens alone.

In accordance with common breeding practices (see [55] and [60] for more detailed explanations), drone producing queens (DPQs) and potential dams of the queens in the next generation (breeding queens, BQs) formed two mutually exclusive groups. A collection of eight DPQs formed a so-called mating station. The 12 drones, which an individual queen mated with, always came from one single mating station. The DPQs on a mating station were simulated to share a common dam BQ. In honeybee breeding theory, such mating stations are often seen as an analogue to sires in other farm animals [49, 52]. In situations where this analogy applies, we therefore refer to them as pseudo sires. The high maintenance effort of secure mating stations in reality leads to relatively small numbers of pseudo sires in honeybee breeding schemes. Our simulations covered the time period of 100 years. We simulated a small population in which 300 BQs and four pseudo sires were created each year, as well as a larger population with 1000 BQs and 10 pseudo sires.

Each simulated year was generally characterized by the following events, which will be further discussed in detail:

- Queen production, including
  - Dam selection
  - Inheritance of true breeding values
- Queen mating
- Colony production and performance tests
- Breeding value estimation for the next year

The last two events occurred only in the second and later years.

The main simulations were implemented in C, while the creation of initial genetic distribution and the statistical analysis of the results were written in R [61]. The source code of the program as well as the output of the simulations and a script for the statistical analyses are stored in Dryad [62].

### Dam selection

**Fig 1:**
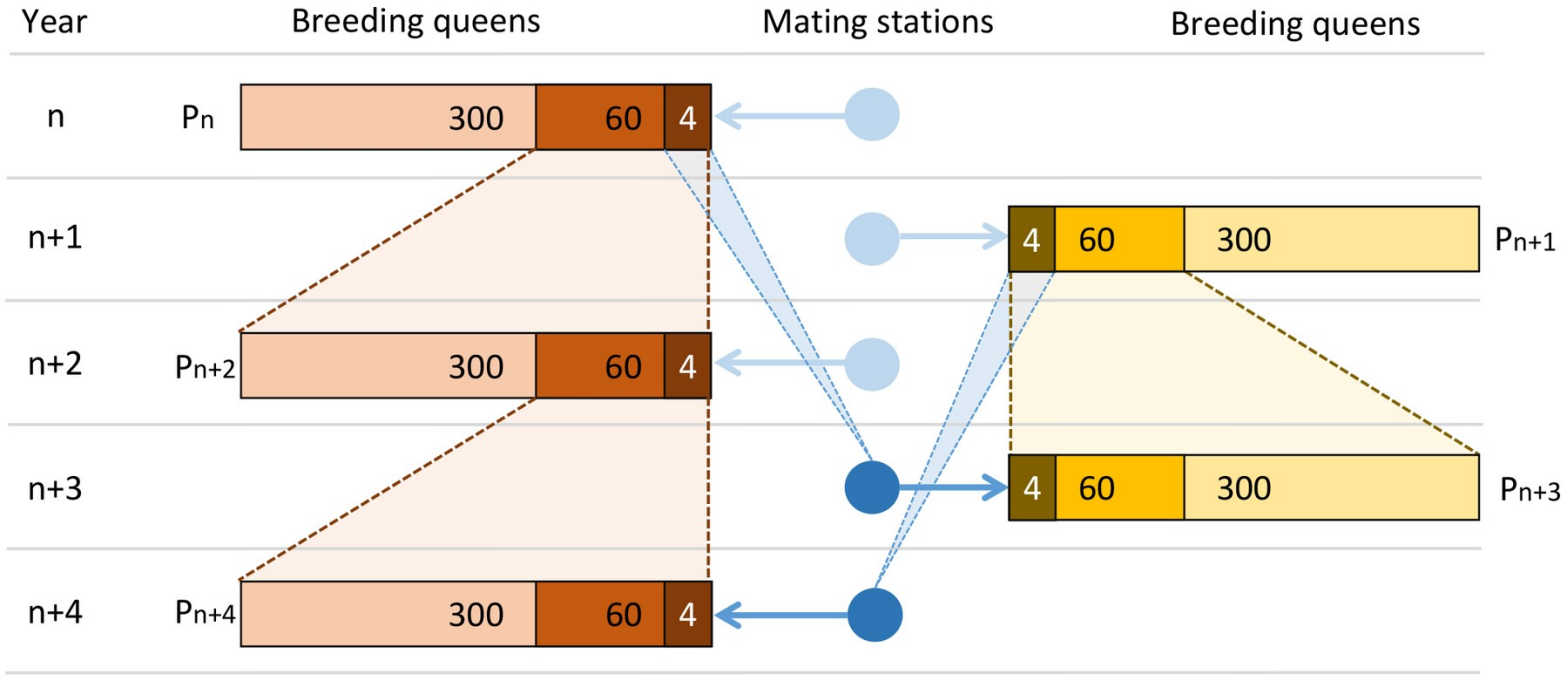
Breeding scheme of the small population. In each year, there are 300 new breeding queens (BQs), of which the best 60 produce the next generation of BQs (generation interval: two years). Each of the four best BQs produce a pseudo sire each year (generation interval: three years). For the larger population, the numbers (300, 60, 4) have to be replaced by (1000, 200, 10).

In each of the first two years, 300 (resp. 1000 in the larger population) BQs were created without specified parents. These base population BQs were assumed to be unrelated. Similarly, in each of the first three years, four (resp. 10) pseudo sires were created without specified parents. Every pseudo sire comprised eight DPQs. No relationships among DPQs or between DPQs and BQs were assumed in the first three years. Beginning in the third year, two-year-old BQs were available, and their colonies had estimated breeding values. With the breeding value estimation following the theory developed in [49], queens and their worker groups had direct and maternal estimated breeding values, where the sum of both breeding values of the worker group constituted the selection criterion (SC) (see [49, 63] for a detailed motivation of the SC). By truncation selection based on the SC, 60 (resp. 200) of the two-year-old BQs were chosen to serve as dams for the next generation of BQs. Each of the selected dams produced five new breeding queens. In the fourth year and each year thereafter, four (resp. 10) three-year-old BQs were selected as dams of the next generation of DPQs. The BQs were chosen by truncation selection based on the SC, and each of them mothered the setup of one mating station (see also Fig 1 for an overview of the breeding scheme).

### Inheritance of true breeding values

The genetics of the bees were simulated according to three different models: (i) a finite locus model with 200 unlinked loci, (ii) a finite locus model with 400 unlinked loci, and (iii) the infinitesimal model. We will refer to the three models as FL200, FL400, and INF, respectively. For the simulations, we decided on a normally distributed trait with an initial direct (worker) additive genetic variance of ${\sigma_{A}^{d}}^{2}=2$, an initial maternal (queen) additive genetic variance of ${\sigma_{A}^{m}}^{2}=1$, and a residual effect with variance $\sigma_{E}^{2}=1$. Two sets of simulations were run with different negative correlations between maternal and direct effects. In the first case, we chose a moderate negative covariance of $\sigma_{A}^{md}=-0.25$, and in the second case, we chose a stronger negative covariance of $\sigma_{A}^{md}=-0.75$. These numbers reflect initial maternal effect heritabilities of $h_{m}^{2}=0.42$, resp. $h_{m}^{2}=0.53$, and direct effect heritabilities of $h_{d}^{2}=0.27$, resp. $h_{d}^{2}=0.34$, with genetic correlations of *r_md_* = −0.18, resp. *r_md_* = −0.53. These relations are in broad accordance with the breeding parameters determined for several traits by various studies [47, 63–65]. We note that as described in [65], the direct effect heritability $h_{d}^{2}$ measures the amount of phenotypic variance that can be attributed to the worker groups. It does not, however, reflect the scope for selection response when selecting virgin queens according to the theory developed in [66]. No dominance or epistatic effects were simulated.

We refer to the matrix of initial genetic parameters as

$$\begin{array}{c} \Sigma_{A}=(\begin{array}{cc} {\sigma_{A}^{d}}^{2} & \sigma_{A}^{md}\\ \sigma_{A}^{md} & {\sigma_{A}^{m}}^{2} \end{array}). \end{array}$$ (1)

The matrix Σ_*A*_ fulfilled three purposes in the simulations.

1. It defined the variance structure of breeding values in the base population.
2. It described the assumed genetic covariances in the BLUP breeding value estimation. (Σ_*A*_ was not updated for this purpose during the simulation, although the genetic parameters shifted due to drift and selection in the finite locus models)
3. It described the variance of Mendelian sampling in the inheritance of breeding values in the infinitesimal model. Also, for this purpose, Σ_*A*_ remained constant up to corrections for parental inbreeding during the simulation because selection does not affect the variance of Mendelian samplings in the infinitesimal model [67, 68].

We further refer to the distinct combinations of the genetic model (FL200, FL400, and INF), genetic correlations (*r_md_* = −0.18 and *r_md_* = −0.53), and population sizes (300 BQs and 1000 BQs per year) by adding the genetic correlations as subscripts and the population sizes as superscripts to the genetic model (cf. Table 1).

**Table 1:** Simulation settings.

population size	genetic correl.	fin. locus, 200 loci	fin. locus, 400 loci	infinitesimal
300 BQs per year	*r*_*md*_ = −0.18	$\text{FL}200_{-0.18}^{300\;\text{BQs}}$	$\text{FL}400_{-0.18}^{300\;\text{BQs}}$	${\text{INF}}_{-0.18}^{300\;\text{BQs}}$
300 BQs per year	*r*_*md*_ = −0.53	$\text{FL}200_{-0.53}^{300\;\text{BQs}}$	$\text{FL}400_{-0.53}^{300\;\text{BQs}}$	${\text{INF}}_{-0.53}^{300\;\text{BQs}}$
1000 BQs per year	*r*_*md*_ = −0.18	$\text{FL}200_{-0.18}^{1000\;\text{BQs}}$	$\text{FL}400_{-0.18}^{1000\;\text{BQs}}$	${\text{INF}}_{-0.18}^{1000\;\text{BQs}}$
1000 BQs per year	*r*_*md*_ = −0.53	$\text{FL}200_{-0.53}^{1000\;\text{BQs}}$	$\text{FL}400_{-0.53}^{1000\;\text{BQs}}$	${\text{INF}}_{-0.53}^{1000\;\text{BQs}}$

Overview of the 12 different simulation settings with respect to the genetic model, population size, and correlation between the queen and worker effect.

#### Finite locus model

In the finite locus models, we assumed the trait to be genetically determined by 200 (FL200), respectively 400 (FL400), unlinked biallelic loci, each possessing a queen and a worker effect. In accordance with [67], we decided to use a U-shaped β(0.5,0.5)-distribution for the allele frequencies in the base population. The distribution of the allele effects is usually assumed to be heavy-tailed. Popular distributions for modelling such effects are gamma and exponential or Laplace distributions [69, 70], while [71] argues for a mixture of distributions. In [15], a mixture between a Laplace and a normal distribution with weights of 0.95 and 0.05 was utilized to describe the effects of the QTL. As this assumption allows for a straightforward generalization to the multivariate case, we also chose a distribution of QTL effects that was dominated by a Laplace distribution.

The genetic setup for the base population was created as follows:

Let *a*_1_ and *a*_2_ be the two possible alleles at a locus with frequencies *p*_1_ and *p*_2_, respectively. Let *n* be the total number of loci. Then the allele effects $E_{1}=(\begin{array}{c} E_{1}^{d}\\ E_{1}^{m} \end{array})$ and $E_{2}=(\begin{array}{c} E_{2}^{d}\\ E_{2}^{m} \end{array})$ of alleles *a*_1_ and *a*_2_ were determined in the following steps:

- Generate a random vector **e** following a distribution
  $$\begin{array}{c} 0.95\cdot L\left(0,\Sigma_{A}\right)+0.05\cdot N\left(0,\Sigma_{A}\right), \end{array}$$ (2)
  where L and N denote the multivariate Laplace and normal distribution, respectively.
- Obtain preliminary allele effects
  $$\begin{array}{ccc} {\tilde{E}}_{1}=\frac{p_{2}-p_{1}+1}{n}e, & & {\tilde{E}}_{2}=\frac{p_{2}-p_{1}-1}{n}e. \end{array}$$ (3)

By this transformation, the expected value for the allele effect at each locus is zero, and the expected additive genetic variance generated by all loci is Σ_*A*_. In a concrete realization, however, the obtained additive genetic variance ${\tilde{\Sigma }}_{A}$ generally differs from Σ_*A*_. Therefore, after the preliminary allele effects were calculated for all loci, we applied a further post-correction step to obtain the allele effects and ensure that the additive genetic variance over all loci is Σ_*A*_.

- Calculate **E**_1_ and **E**_2_ as
  $$\begin{array}{c} E_{1,2}=\Sigma_{A}^{\frac{1}{2}}{\tilde{\Sigma }}_{A}^{-\frac{1}{2}}{\tilde{E}}_{1,2}. \end{array}$$ (4)

The script for the creation of these possible alleles at the QTL was written in R [61] using the packages MASS [72] and L1pack [73] to create multivariate normal and Laplace distributed vectors, respectively [62]. Once the setup of possible alleles was generated, the queens of the base population were each equipped with two alleles at each locus, where the alleles were randomly drawn based on the specified frequencies. Queens of later generations inherited their genes by receiving one of the two alleles of the dam and the single allele of the sire drone at each gene locus. Drones inherited one of the two alleles of the dam at random. No mutations were modelled in the inheritance of alleles. For an individual queen, its true breeding value (TBV) was calculated as the sum of the allele effects of its whole genome. To obtain comparable values for the TBV of haploid drones and diploid queens, the true breeding value of a drone was defined as twice the sum of the allele effects of its genome. This matches the interpretation of drones as diploid but homozygous at each locus as described in [74]. The worker group *W* of a queen *Q* received as its breeding value the mean value of *Q*’s TBV and the average TBV of the drones that *Q* mated with, as shown in the following equation:

$$\begin{array}{c} {\text{TBV}}_{W}=\frac{1}{2}({\text{TBV}}_{Q}+{\bar{\text{TBV}}}_{D}) \end{array}$$ (5)

#### Infinitesimal model

In the infinitesimal model (INF), all queens from the base population received their true breeding values as realizations of a normally distributed random vector with expectation (0, 0)′ and variance Σ_*A*_, as explained in [75]. The inheritance of true breeding values from parents to offspring followed the theory of [76] with necessary adaptations to the haploidy of drones. In particular, the inheritance of the TBV from a breeding queen *B* and a drone *D* to a daughter queen *Q* was realized as

$$\begin{array}{c} {\text{TBV}}_{Q}=\frac{1}{2}({\text{TBV}}_{B}+{\text{TBV}}_{D}+\sqrt{1-F_{B}}\cdot \Phi ), \end{array}$$ (6)

where the Mendelian sampling Φ was the realization of a normally distributed random vector with expectation (0, 0)′, and variance Σ_*A*_. *F*_*B*_ denotes the inbreeding coefficient of *B*. *In vivo*, exact estimations of relationships and inbreeding coefficients are difficult to obtain because the relatedness of two offspring of the same dam can vary between 0.25 and 0.75 [52]. However, *in silico*, drones were simulated individually, so we could keep track of the exact relationships between siblings and, consequently, between all individuals. The calculation of relationships was performed as stated in [77]. Therefore, at this point, the simulations did not need to rely on approximated calculations of *F*_*B*_ as described in [52] or [49, 59]. The inheritance from a DPQ *Q* to a drone *D* was realized as

$$\begin{array}{c} {\text{TBV}}_{D}={\text{TBV}}_{Q}+\sqrt{1-F_{Q}}\cdot \Phi , \end{array}$$ (7)

where again the Mendelian sampling Φ was the realization of a normally distributed random vector with expectation (0, 0)′ and variance Σ_*A*_. The worker group *W* of a queen *Q* received its breeding value as in the finite locus models according to Eq 5.

### Queen mating

When all queens of a year were created, the BQs were assorted to the pseudo sires. BQs with the same dam were sent to the same mating station. The distribution of the sister groups to the pseudo sires was random. When a queen was assigned to a pseudo sire, 12 drones were produced, where the dam of each drone was chosen at random among the 8 DPQs of the mating station. The data of the drones (alleles, in the case of the finite locus models, and TBV, in the case of the infinitesimal model) were henceforth stored together with the queen data.

### Colony production and performance tests

After the mating of the newly-born BQs, the one-year-old BQs were assigned a colony. The colony received true breeding values according to Eq 5. The simulated performance of a colony was calculated as the sum of the true maternal breeding value of the queen (${\text{TBV}}_{Q}^{m}$), the true direct breeding value of the worker group (${\text{TBV}}_{W}^{d}$), and a random residual effect being the realization of a normally distributed random variable with a mean of zero and variance of $\sigma_{E}^{2}$. As the sum ${\text{TBV}}_{Q}^{m}+{\text{TBV}}_{W}^{d}$ determines the genetic share of the outcome of the performance test, the main aim of the selection is to maximize this value over time. Therefore, we refer to this sum as the performance criterion (PC).

Subsequently, a BLUP breeding value estimation was carried out using the BLUPF90 software [78] with the relationships between BQs and colonies calculated as described in [59]. The breeding value estimation was based on the parameters $\sigma_{E}^{2}=1$ and Σ_*A*_ as specified in Eq 1. No parameter estimation was carried out within the simulations, and the BLUP parameters were not adjusted to represent genetic changes due to drift and selection in settings FL200 and FL400.

### Repetitions

Simulations using the INF model were repeated 100 times to obtain reliable results. In the simulations with a finite number of loci, we created 10 sets of allele frequencies and allele effects for each of the finite locus settings. With each of these sets, simulations for both population sizes were repeated 100 times.

*A posteriori*, we did an investigation on the repetitions needed for future simulations based on the variance of the outcomes within the respective repetitions. Following [79], we estimated the number of needed repetitions of the simulations from the variances in the results. If the standard deviation of the result of the repetitions is *s*, and the tolerated error in the estimation for the mean value is *e*, then, with a minimum repetition number of

$$\begin{array}{c} N_{\text{min}}={\left(\frac{s\cdot z_{1-\frac{\alpha }{2}}}{e}\right)}^{2}, \end{array}$$ (8)

there is a security level of 1 − *α* that the calculated mean value differs from the true expectation by less than *e*. Here, *z* refers to the quantiles of the standard normal distribution.

As the outcomes of the simulations depend on the chosen model and deviations between simulation results, and *in vivo* observations due to model inaccuracy are possible, we decided to allow for an error rate of up to 10%. We further investigated how many repetitions are needed to fall below this error rate with a confidence level of 99%.

## Results

### Genetic gain

Table 2 summarizes the average accumulated genetic gain in the breeding colonies. The genetic gain for the performance criterion (PC) after 100 years ranged from 14.90 units in model $\text{FL}200_{-0.53}^{300\;\text{BQs}}$ to 41.37 units in model ${\text{INF}}_{-0.18}^{1000\;\text{BQs}}$. In all settings, the genetic value for the direct effect increased more than that of the maternal effect. This remained the case when we corrected for the different genetic standard deviations of maternal and direct effects. The ratio between genetic gain for direct and maternal effects after 100 years ranged from 1.58 ($\text{FL}400_{-0.18}^{300\;\text{BQs}}$) to 2.81 ($\text{FL}400_{-0.53}^{1000\;\text{BQs}}$) before correction and 1.12 ($\text{FL}400_{-0.18}^{300\;\text{BQs}}$) to 1.99 ($\text{FL}400_{-0.53}^{1000\;\text{BQs}}$) after correction.

**Table 2:** Genetic gain.

*r*_*md*_	-0.53						-0.18					
model	FL200		FL400		INF		FL200		FL400		INF	
BQs per year	300	1000	300	1000	300	1000	300	1000	300	1000	300	1000
	before correction for different genetic standard deviations											
	after 20 years											
maternal effects	1.88	1.77	1.85	1.73	1.93	1.74	2.99	3.03	3.01	3.05	2.99	3.10
direct effects	3.18	3.73	3.31	3.91	3.33	3.96	4.67	5.18	4.67	5.18	4.76	5.30
performance criterion	5.08	5.54	5.18	5.69	5.26	5.75	7.69	8.27	7.74	8.30	7.80	8.46
	after 100 years											
maternal effects	5.27	4.96	5.57	5.22	8.09	7.94	8.28	8.57	9.99	10.52	13.67	14.71
direct effects	9.62	11.73	11.84	14.66	15.96	20.14	13.37	15.26	15.78	18.22	22.54	26.60
performance criterion	14.90	16.70	17.40	19.88	24.08	28.14	21.65	23.83	25.79	28.76	36.23	41.37
	after correction for different genetic standard deviations											
	after 20 years											
maternal effects	1.88	1.77	1.85	1.73	1.93	1.74	2.99	3.03	3.01	3.05	2.99	3.10
direct effects	2.25	2.64	2.34	2.77	2.36	2.80	3.30	3.66	3.30	3.66	3.36	3.75
performance criterion	3.38	3.69	3.45	3.80	3.51	3.83	4.64	4.99	4.67	5.01	4.70	5.10
	after 100 years											
maternal effects	5.27	4.96	5.57	5.22	8.09	7.94	8.28	8.57	9.99	10.52	13.67	14.71
direct effects	6.80	8.29	8.37	10.36	11.29	14.24	9.45	10.79	11.16	12.89	15.94	18.81
performance criterion	9.93	11.13	11.60	13.25	16.05	18.76	13.05	14.37	15.55	17.34	21.85	24.95

The average genetic gain in the different models after 20 and 100 years. Maternal and direct effects were taken from the BQs of that year; PC denotes the performance criterion. In the lower part of the table, values are divided by the the respective initial genetic standard deviations to improve their comparability.

**Fig 2:**
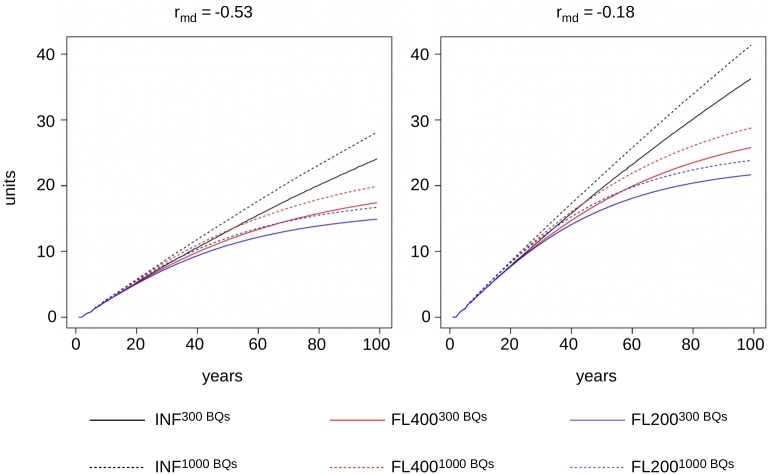
Genetic gain in the performance criterion. Development of the genetic gain in the performance criterion (PC) over the course of 100 years in the models INF (black), FL400 (red), and FL200 (blue) for population sizes consisting of 300 breeding queens (solid lines) and 1000 breeding queens (dashed lines), respectively. Results are shown for a strong negative correlation between maternal and direct effects, *r*_*md*_ = −0.53, (left hand side) and a weak negative correlation, *r*_*md*_ = −0.18 (right hand side).

Throughout all settings, the genetic values for the worker groups were, on average, slightly higher than those for the corresponding queens. The models with strong negative correlations showed a lower genetic gain than the corresponding model with a low negative correlation. This remained true when we corrected the genetic gain by the different genetic standard deviations. A larger population size had a slight positive effect on genetic gain. The ratio between the genetic gains for different population sizes in the PC after 100 years ranged between 1.10 (FL200_−0.18_) and 1.17 (INF_−0.53_). In the long-term, model INF always predicted the highest genetic gain, followed by FL400 and FL200, in this order. The ratio between genetic gain in the PC under model INF and model FL200 ranged between 1.62 (300 BQs, *r*_*md*_ = −0.53), and 1.74 (1000 BQs, *r*_*md*_ = −0.18) after 100 years. After 20 years, this ratio only ranged between 1.013 (300 BQs, *r*_*md*_ = −0.18) and 1.038 (1000 BQs, *r*_*md*_ = −0.53). Genetic gain in model INF showed an almost linear increase, whereas models FL200 and FL400 showed clearly sublinear behavior after 20 to 25 years (cf. Fig 2).

### Genetic variance and inbreeding

**Fig 3:**
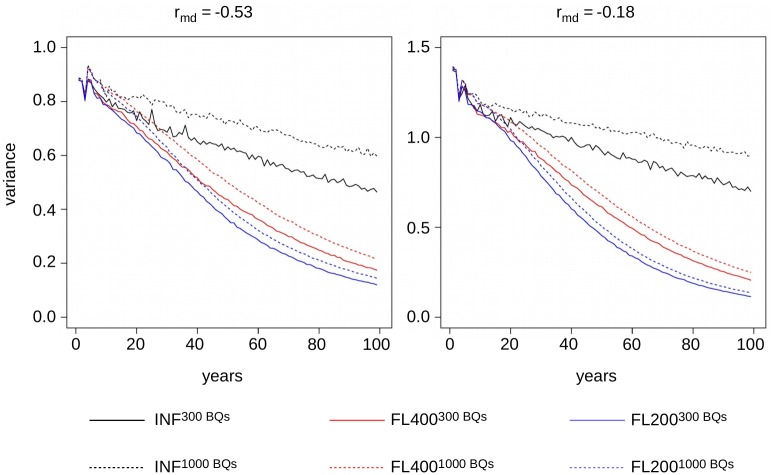
Additive genetic variance in the performance criterion. Development of the additive genetic variance in the performance criterion (PC) over the course of 100 years in the models INF (black), FL400 (red), and FL200 (blue) for population sizes consisting of 300 breeding queens (solid lines) and 1000 breeding queens (dashed lines), respectively. Results are shown for a strong negative correlation between maternal and direct effects *r*_*md*_ = −0.53, (left hand side) and a weak negative correlation *r*_*md*_ = −0.18 (right hand side).

The development of the mean inbreeding coefficients of the breeding queens depended mainly on the population size. After 100 years, the average inbreeding coefficient in the small populations was between 0.37 (model $\text{FL}200_{-0.18}^{300\;\text{BQs}}$) and 0.43 (model ${\text{INF}}_{-0.53}^{300\;\text{BQs}}$). The corresponding values in the larger populations were 0.24 ($\text{FL}200_{-0.18}^{300\;\text{BQs}}$) and 0.30 (${\text{INF}}_{-0.53}^{1000\;\text{BQs}}$). This means there were yearly increases of about Δ*F* = 0.004 and Δ*F* = 0.003, respectively. However, the development of genetic diversity in the population depended much more on the underlying genetic model than on the population size. All simulations showed an initial decrease in genetic variance due to the Bulmer effect [68], but after 100 years, only the finite locus models showed a tremendous decay in genetic variance (cf. Fig 3). After 100 years, the variances in the PC in the INF model only went down to 52% for the small population and to between 64% and 67% for the larger population. The corresponding values for the finite locus models ranged from 8.3% ($\text{FL}200_{-0.18}^{300\;\text{BQs}}$) to 24.4% ($\text{FL}400_{-0.53}^{1000\;\text{BQs}}$). The ratio between genetic variance in the PC under the model INF and model FL200 ranged between 3.88 (300 BQs, *r_md_* = −0.53) and 6.58 (1000 BQs, *r_md_* = −0.18) after 100 years. After 20 years, this ratio only ranged between 1.07 (300 BQs, *r_md_* = −0.53) and 1.13 (300 BQs, *r_md_* = −0.18).

### Variance in the results

Since all simulations were Monte Carlo simulations, variance could be observed in the results. In models FL200 and FL400, there were two sources of variance. One source was the 10 different distributions of allele effects, and the other source was the 100 repetitions that were carried out with each distribution. In model INF, only the latter source of variance was present. The standard deviations in the results with a fixed distribution of allele effects after 20 and 100 years are shown in Table 3. When we compare these standard deviations with the realized mean values in the genetic gain, we find a relatively high standard deviation of up to 25% in the maternal true breeding values, whereas the standard deviation in the PC is relatively low (under 10%). When we consider the standard deviations in the genetic variance, we obtain high percentages for the PC (31.1% in model $\text{FL}200_{-0.53}^{300\;\text{BQs}}$) and slightly lower percentages of around 20% for the maternal and direct true breeding values. In the models FL200 and FL400, the different distributions of allele effects mostly had only small influences of under 10% on genetic gain and variance.

**Table 3:** Genetic gain.

*r*_*md*_	-0.53						-0.18					
model	FL200		FL400		INF		FL200		FL400		INF	
BQs per year	300	1000	300	1000	300	1000	300	1000	300	1000	300	1000
	population mean											
	after 20 years											
maternal effects	0.466	0.347	0.441	0.346	0.446	0.326	0.404	0.299	0.394	0.294	0.407	0.333
direct effects	0.602	0.451	0.611	0.446	0.618	0.390	0.562	0.389	0.557	0.401	0.533	0.392
performance criterion	0.458	0.346	0.489	0.358	0.547	0.342	0.547	0.375	0.531	0.381	0.598	0.397
	after 100 years											
maternal effects	0.776	0.596	0.829	0.681	0.901	0.700	0.745	0.595	0.734	0.600	0.879	0.711
direct effects	1.001	0.827	1.064	0.913	1.178	0.889	0.980	0.813	0.989	0.820	1.103	0.812
performance criterion	0.727	0.583	0.774	0.671	0.884	0.658	0.966	0.830	0.961	0.780	0.844	0.701
	population variance											
	after 20 years											
maternal effects	0.126	0.096	0.118	0.090	0.113	0.102	0.123	0.086	0.111	0.078	0.110	0.074
direct effects	0.222	0.156	0.215	0.150	0.196	0.139	0.214	0.148	0.199	0.138	0.190	0.119
performance criterion	0.129	0.097	0.125	0.092	0.125	0.097	0.151	0.110	0.151	0.110	0.135	0.096
	after 100 years											
maternal effects	0.066	0.058	0.066	0.060	0.067	0.055	0.052	0.047	0.051	0.048	0.071	0.059
direct effects	0.110	0.082	0.113	0.094	0.119	0.093	0.085	0.072	0.085	0.069	0.148	0.092
performance criterion	0.037	0.032	0.049	0.042	0.091	0.062	0.033	0.029	0.045	0.040	0.089	0.077

Standard deviations in the outcomes of the simulations with a fixed setting. Maternal and direct effect were taken among the BQ of that year. PC denotes the performance criterion.

### Bias in estimated breeding values

**Fig 4:**
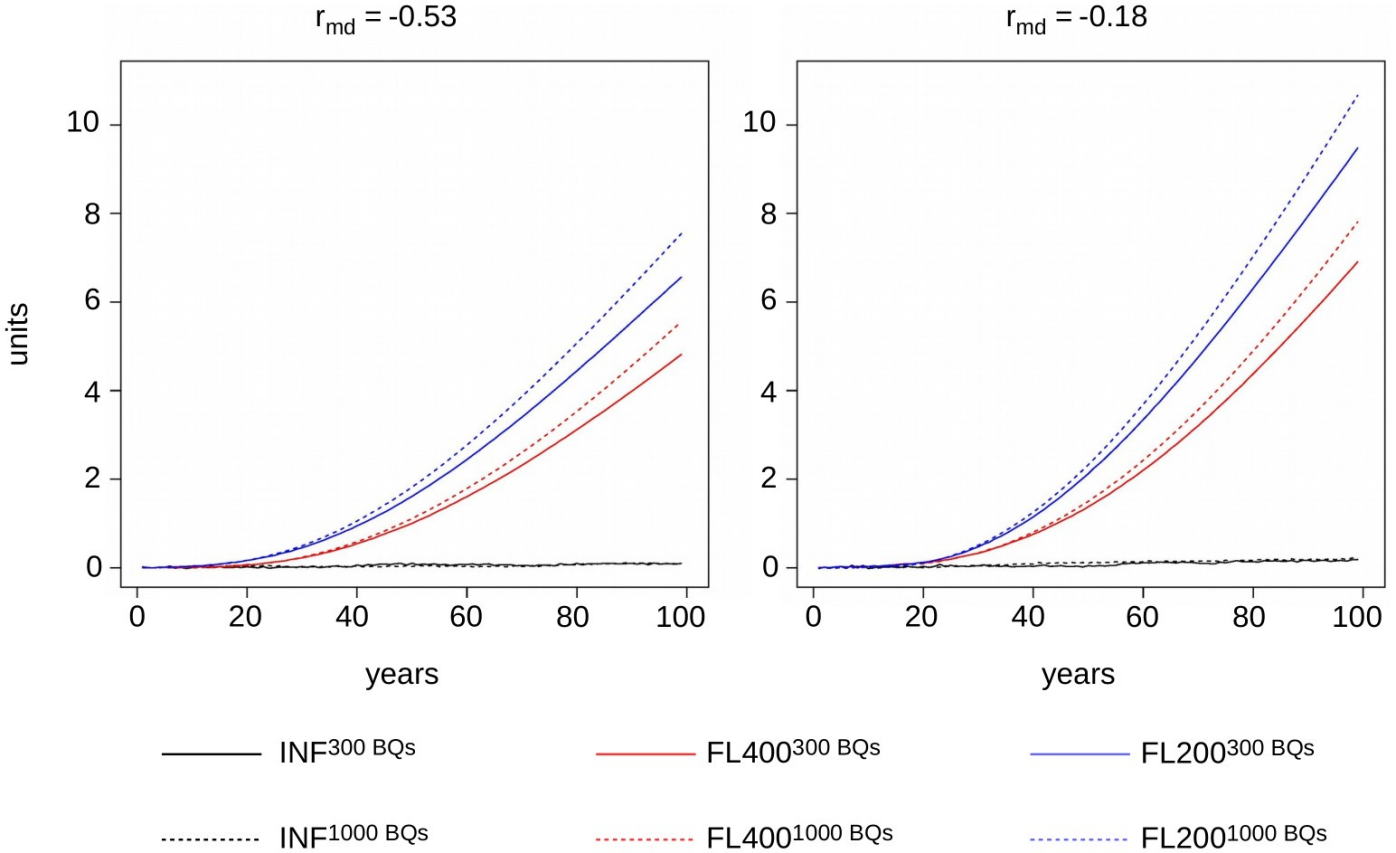
Bias of estimated breeding values. Development of the bias of estimated breeding values in the selection criterion (SC) over the course of 100 years in the models INF (black), FL400 (red), and FL200 (blue) for population sizes consisting of 300 breeding queens (solid lines) and 1000 breeding queens (dashed lines), respectively. Results are shown for a strong negative correlation between maternal and direct effects, *r*_*md*_ = −0.53, (left hand side) and a weak negative correlation, *r*_*md*_ = −0.18 (right hand side).

When we compared the estimated BLUP breeding values of individuals with their true breeding values, we saw no bias in the simulations based on model INF. The estimated breeding values in the settings FL200 and FL400, however, developed an increasing bias over time (cf. Fig 4). After 100 years of selection, the ratios between estimated and true breeding values for the selection criterion SC ranged from 1.27 in setting $\text{FL}400_{-0.18}^{300\;\text{BQs}}$ to 1.45 in setting $\text{FL}200_{-0.53}^{1000\;\text{BQs}}$.

## Discussion

### Model selection

In the finite locus model, all loci were assumed to be potentially pleiotropic, whereas, in reality, many loci will only contribute to either direct or maternal effects. Works by Guo et al. [29] and Gupta et al. [41] consequently proposed models where only a part of the loci was pleiotrophic. In contrast, [29] and [41] proposed models where many of the loci contribute to either the direct or maternal effects. As a tradeoff, however, [29] had to assume that all pleiotrophic loci have the exact same contribution on every trait, which seams unrealistic. In [41], the genetic covariance between direct and maternal effects could not be defined *a priori* but only determined *a posteriori* once a base population was created.

Furthermore, neither of the approaches in [29, 41] allow for a straightforward generalization for arbitrary multiple trait models. Our choice of the multivariate Laplace distribution was made because it allows for natural generalizations to multiple trait models and is in line with common one-dimensional assumptions, such as the QTL effects following a heavy-tailed distribution [15]. The strong negative correlation between direct and maternal effects observed in the honeybee [47, 63–65] indicates that in this species, many loci are indeed pleiotrophic or loci with direct and maternal effects that are closely linked with each other. An *a posteriori* investigation of the simulated allele effects showed that although all loci were potentially pleiotrophic, about a fourth of the loci showed practically only one effect, with minor differences in the different genetic setups. See S1 Appendix for details.

Nevertheless, detailed investigations with genetic data resembling reality as closely as possible are desirable, especially when more honeybee-specific information is available in the future due to the introduction of high definition SNP-chips for the honeybee [80]. Based on the explanations given above, we expect that gene loci will prove more heterogeneous than assumed in our models. Therefore, the assumptions of the infinitesimal model will be violated to an even greater extent. Hence, it is to be expected that the results will show an even greater deviation. With more knowledge about the properties of specific traits, it will also be possible to further investigate the interplay of different traits with their direct and maternal expressions, as well as nonadditive effects.

To our knowledge, there are no suitable estimates for the number of loci that control the various traits in honeybee breeding. In other species, the estimated number of QTLs responsible for a trait has increased drastically within the last two decades, to numbers above 2000 [81]. Hence, the numbers of 200 and 400 loci that we chose are rather conservative. They are, however, in line with the findings and assumptions of [70] for a swine population, as well as [41] for the honeybee.

The linkage of gene loci has an influence on the accuracy of BLUP-based breeding value estimation [82]. This effect is due to the fact that linkage shrinks the effective number of loci responsible for a trait [83, 84]. As all loci in our simulations were considered unlinked, this also justifies the relatively small number of QTL. However, linkage may diminish the rate of drift and, therefore, lead to a smaller bias in breeding value estimation. We conducted a small study based on setting $\text{FL}400_{-0.18}^{300\;\text{BQs}}$ in which we included linkage between the loci based on the honeybee genome but found only minor differences from the simulations without linkage. Details on this smaller-scale study are given in S2 Appendix.

While linkage does not play a major role in our simulation, it must necessarily be included in simulation studies that rely on genomic selection strategies [40, 41]. If such simulations are carried out for the honeybee, one should account for the high recombination rate in this species [85–87]. Linkage effects and the high recombination rate will also play a role if future simulations focus on the sex-determining locus [88].

### Comparison of the models

#### Direct and maternal genetic gain

The overall findings in genetic development with the close-to-linear genetic gain in model INF and sublinear behavior in the finite locus models is in line with existing simulation studies [19, 89].

Linksvayer and Wade [90, 91] showed that under natural selection, fitness traits that depend on the genetic properties of the worker group have a reduced selection potential compared to fitness traits that depend on the genetic properties of a queen. However, as by the definition of the selection criterion only the estimated (direct and maternal) breeding values of the worker group are taken into consideration for the breeding decisions, the approaches of [90, 91] cannot explain different developments of direct and maternal effects. The negative correlation between direct and maternal effects has been long known to complicate breeding decisions and, when it is not taken care of correctly, it can even cause the selection to run in the opposite direction of what was intended [92]. The observation that the direct effects underwent a larger genetic gain than the maternal effects, also relative to the respective additive variances, can be explained as follows. As the direct effects had a higher genetic variance than the maternal effects and both effects are negatively correlated, in many alleles with adverse direct and maternal effects, the direct outweighed the maternal component. At these loci, selection was therefore directed actively against the maternal effects which caused the different developments. Another factor that influences the genetic development is the accuracy of breeding value estimation. In our simulations, the correlation between true and estimated breeding values of the worker groups were slightly higher for the maternal effects (between 0.54 and 0.65, depending on the simulation setting) than for the direct effects (between 0.46 and 0.57). These differences were too small to impact the preference for direct effects significantly. However, with equal additive genetic variances of direct and maternal effects, the improved estimation accuracy will cause a stronger selection for maternal effects. A small simulation study with varying additive genetic variances for the direct and maternal effects confirms these theoretical considerations. It is included in S3 Appendix. Likewise, the simulations in [35, 36] also assumed higher direct than maternal genetic variances and predict a main selection focus on the direct effects. These studies even prognosticate a negative selection of the maternal effects when an even stronger negative correlation with the direct effects occurs. Further simulation studies that shed more light on the development and consequences of the negative correlation between direct and maternal effects under selection and genetic drift are highly desirable.

#### Development of genetic variance

When it comes to genetic variance, the observed drastic decay in the finite locus models matches the findings of [18]. We ascribe the lower genetic gain and variance in the finite locus models mainly to the inhomogeneity of the allele effects. Alleles with a higher impact on the selection trait have the highest contribution to the genetic variance but are also the genes that are mainly selected for. Therefore, in a finite locus model where few loci contribute to a large amount of the genetic variance, it is precisely the favorable alleles at these loci that have the highest tendency to become fixed within the population [93]. The larger population could preserve more genetic variance over time and could, therefore, maintain a more sustainable genetic gain. This is in line with earlier simulation studies [19, 94]. However, different population sizes showed only minor differences in the development of genetic variance. We conclude that the decay of genetic variability is mainly caused by the selection intensity and that genetic drift and the accumulation of inbreeding play only a smaller role as these are clearly influenced by the effective population size. We expect that lowering the ratio of BQs to pseudo sires could improve the preservation of genetic variance as this would immensely weaken the selection intensity on the paternal side. Such a measure is likely to decrease genetic gain in the short-term, but in the long-term, it may be profitable [94, 95]. Differences in the outcomes of the three models suggest that in simulations exceeding 20 years (i.e., 10 generations on the female and seven generations on the male side), the genetic setup has to be chosen carefully.

Finite locus models, as we implemented them, fail to explain the phenomenon of ‘missing heritability’ [96]. This term describes the observation that the additive genetic variance in several quantitative traits can only be partially explained by the QTL associated with this trait. Consequently, [97] suggests relying on the infinitesimal model despite its biologically incorrect assumptions. In [97], the author provides examples of long-term selection in poultry that showed a linear development over a span of 50 generations and beyond. In contrast, [98] showed a substantial loss in genetic variance in a selection experiment for low mating activity in Japanese quail, and [99] succeeded in explaining most of the additive genetic variance in complex traits in cattle by genetic markers. Without further knowledge about the actual genetic architecture of the simulated trait, one will always bear the risk that long-term simulation studies based on the infinitesimal model are likely to give overly optimistic predictions on the conservation of genetic variance. However, predictions based on finite locus models might be too pessimistic. An interesting field of research appears to be the question of how non-additive genetic effects (dominance, epistasis) can support the maintenance of additive genetic variance in the finite locus model [15].

#### Variance in the results

One possible explanation for the relatively high variance in the results for the average maternal breeding value of the population is that selection focused more on the direct trait component, and the selection of the maternal part of the trait was, therefore, less stringent. However, further investigation of this finding is necessary.

The required repetitions for secure results that we calculated via Eq 8 can be found in Table 4. They are all well below 100, so our simulation setup was adequate. The different realized distributions of allele effects within a finite locus model showed only minor effects on the outcomes. We conclude that a single realization will suffice for many purposes and that more diverse results are to be expected with the introduction of more distributions rather than more realizations of the same distribution [12, 18].

**Table 4:** Required repetitions.

*r*_*md*_	-0.53						-0.18					
model	FL200		FL400		INF		FL200		FL400		INF	
BQs per year	300	1000	300	1000	300	1000	300	1000	300	1000	300	1000
	population mean											
	after 20 years											
maternal effects	42	26	38	27	36	24	13	7	12	7	13	8
direct effects	24	10	23	9	23	7	10	4	10	5	9	4
performance criterion	6	3	6	3	8	3	4	2	4	2	4	2
	after 100 years											
maternal effects	15	10	15	12	9	6	6	4	4	3	3	2
direct effects	8	4	6	3	4	2	4	3	3	2	2	1
performance criterion	2	1	2	1	1	1	2	1	1	1	1	1
	population variance											
	after 20 years											
maternal effects	17	9	15	8	13	9	18	8	14	6	13	5
direct effects	15	7	12	6	10	5	14	7	12	6	10	4
performance criterion	25	12	21	10	20	10	16	8	15	7	10	5
	after 100 years											
maternal effects	54	30	34	19	12	5	63	37	29	17	13	6
direct effects	57	28	32	17	10	4	59	38	27	14	14	4
performance criterion	67	34	53	26	26	8	57	33	32	18	11	5

Numbers of repetitions required for a simulation to have a 99% confidence level that the deviation is less than 10%.

#### Estimated breeding values

In the INF model, it was to be expected that no bias would occur in the estimated breeding values since BLUP is an unbiased estimator for breeding values in this model [100]. In finite locus models, however, a shift occurs in allele frequencies, leading to a reduction of heritabilities. As BLUP was run relying on the initial genetic parameters throughout the simulations, the estimated breeding values were positively biased in these cases. The effects of the biased estimates for heritabilities of the genetic response have been shown to be small, while inbreeding would probably have increased even more drastically if BLUP was run with the correct parameters [101]. The simulations show the need in real life breeding to re-estimate genetic parameters regularly unless one explicitly wants to make use of the positive effects of biased parameter estimates on the development of inbreeding as described in [101].

### Honeybees

Our methodological approach and the results are usable for all livestock species. In our opinion, considerations of sustainable long-term breeding concepts have not yet been adequately addressed. This applies, in particular, to the honeybee as the complementary sex determination makes this species especially vulnerable to inbreeding depression [48]. Therefore, a strong focus on the maintenance of genetic diversity is mandatory. Moreover, one has to consider that the replacement of native subspecies by two selected races has reached a degree that leaves many subspecies threatened by extinction [53]. The EU project SmartBees [102] aims to establish breeding strategies for these subspecies, which are well adapted to their regional environmental conditions, to comply with the breeders’ productivity requirements. With their main purpose being the conservation of subspecies, these breeding strategies must be sustainable despite small effective population sizes. The methods we developed here are a good foundation to address such questions.

Unfortunately, the chosen example with 300 colonies and four pseudo sires is representative for some subspecies, such as the Maltese honeybee *Apis mellifera ruttneri* [103] or the Sicilian honeybee *A. m. siciliana* [104]. They need breeding approaches that are substantially different from those of the large breeding population of *A. m. carnica* with 8000 colonies per year, which has shown a strong response to selection within the last two decades [105]. The simulations indicate that the chosen breeding schemes, which we initially saw as realistic, are too strict and will potentially harm any population of honeybees. For practical purposes, other breeding strategies with more pseudo sires or a reduction in the number of offspring within one family, according to [106], will have to be developed. A transfer of the Optimum Contribution Selection [107] to the haplo-diploid case also seems promising.

## Conclusion

We conclude that future long-term simulation studies that design strategies in conservational animal breeding should focus on finite locus models rather than the infinitesimal model to minimize the risk of overly optimistic prognoses. Differences in the outcomes of simulations of type FL200 and FL400 indicate the need for more accurate information on the number and distribution of QTL. Of course, a simulation output of 100 years should always be seen as a reference timeframe as the breeding infrastructure is likely to undergo major changes in this time. Currently, the introduction of genomic selection for the honeybee is assumed to give rise to new breeding strategies [40, 41, 60]. For short-term simulation studies of less than 10 generations, all genetic models appear to be equally feasible.

## Supporting information

S1 Appendix QTL effect analysis. Analysis on the realized distribution of QTL effects.
(PDF)

S2 Appendix Linkage simulations. Small-scale study on the effect of linkage on the results of the simulations.
(PDF)

S3 Appendix Selection strength on direct and maternal effects. Small-scale study on the selection response to direct and maternal genetic effects dependent on the ratio of additive genetic variances.
(PDF)
