# Supplementary material for: Comparison of infinitesimal and finite locus models for long-term breeding simulations with direct and maternal effects at the example of honeybees
Source: PLoS One. 2019 Mar 6;14(3):e0213270. doi: 10.1371/journal.pone.0213270 (PMC6402681; doi:10.1371/journal.pone.0213270)
Supplement: S1 Appendix — Analysis on the realized distribution of QTL effects. (PDF) [file pone.0213270.s001.pdf]

QTL effect analysis

For our simulation study, we generated ten different distributions of allele frequencies and QTL effects for each of the eight settings $\mathrm{FL200}^{300\ \mathrm{BQs}}_{\text{-}0.18}$, $\mathrm{FL200}^{300\ \mathrm{BQs}}_{\text{-}0.53}$, $\mathrm{FL200}^{1000\ \mathrm{BQs}}_{\text{-}0.18}$, $\mathrm{FL200}^{1000\ \mathrm{BQs}}_{\text{-}0.53}$, $\mathrm{FL400}^{300\ \mathrm{BQs}}_{\text{-}0.18}$, $\mathrm{FL400}^{300\ \mathrm{BQs}}_{\text{-}0.53}$, $\mathrm{FL400}^{1000\ \mathrm{BQs}}_{\text{-}0.18}$, and $\mathrm{FL400}^{1000\ \mathrm{BQs}}_{\text{-}0.53}$. The maternal and direct QTL-effects followed a distribution that was dominated by a multivariate Laplace distribution. Therefore, all gene loci were potentially pleiotrophic regarding direct and maternal effects. In reality, however, there will probably be many loci, that only have influence on either the maternal or the direct effect. Therefore we wanted to investigate, how many of the simulated loci actually proved to be pleiotrophic.

For this purpose, we divided the loci into three categories regarding maternal effects and three categories regarding direct effect, leading to nine cross categories: A locus could have a definite effect, an unclear effect or practically no effect maternally and directly.

For the direct effect, the loci were categorized as follows: For a locus l, we let $E^d_{l,1}$, $E^d_{l,2}$ be the direct effects of the two possible alleles at this locus. Then the range of possible direct contribution of the locus l is

$$R^d_l := |E^d_{l,1} - E^d_{l,2}|.$$

Let n be the total number of loci and let the loci be numbered from 1 to n so that $R^d_i \leq R^d_j$ for $i < j$. Then

$$R^d := \sum_{l=1}^{n} |E^d_{l,1} - E^d_{l,2}|$$

describes the total range of possible true direct breeding values. Let n_1 be maximal such that

$$\sum_{l=1}^{n_1} |E^d_{l,1} - E^d_{l,2}| < 0.05 \cdot R,$$

then we considered the loci with the numbers 1 to n_1 to have practically no direct effect.

Conversely, we considered the loci l with

$$|E^d_{l,1} - E^d_{l,2}| > \frac{1}{2n} \cdot R$$

to definitely have a direct effect. All other loci were considered to have an unclear direct effect. For the maternal effect we applied the same definitions. This allowed us to consider these loci as not pleiotrophic that definitely had a direct effect but practically no maternal effect or vice versa. Table 1 gives a survey of the relative frequencies of alleles inside the nine cross categories.

Table 1. Allele frequencies

				maternal effect		
r_{md}	number of loci			practically no	unclear	defititive
-0.18	200	direct effect	practically no	12.1%	3.45%	14.2%
			unclear	3.3%	1.0%	6.15%
			definitive	13.95%	5.9%	39.95%
	400	direct effect	practically no	12.3%	3.4%	13.28%
			unclear	3.45%	1.18%	4.93%
			definitive	13.43%	5.65%	43.15%
-0.53	200	direct effect	practically no	13.9%	3.4%	12.25%
			unclear	3.15%	0.9%	4.8%
			definitive	12.9%	5.55%	43.15%
	400	direct effect	practically no	14.18%	3.68%	12.08%
			unclear	3.3%	0.98%	4.55%
			definitive	12.33%	5.23%	43.7%

Frequencies of alleles in different categories for direct and maternal effects.

With little deviances for the different models we found that only slightly more than 40% of the loci turned out clearly pleiotrophic. About 13% of the loci carried practically no effect at all, while 13% had only a maternal effect and another 13% had only a direct effect. For the rest of the loci it could not be clearly decided if they have a maternal or a direct effect.
