# Supplementary material for: Comparison of infinitesimal and finite locus models for long-term breeding simulations with direct and maternal effects at the example of honeybees
Source: PLoS One. 2019 Mar 6;14(3):e0213270. doi: 10.1371/journal.pone.0213270 (PMC6402681; doi:10.1371/journal.pone.0213270)
Supplement: S2 Appendix — Small-scale study on the effect of linkage on the results of the simulations. (PDF) [file pone.0213270.s002.pdf]

Linkage simulations

Our main simulation studies used unlinked loci in the finite locus models. In order to investigate if the assumption of unlinkedness caused a substantial bias to our results, we also performed a small scale study using linked loci. For this purpose, we generated a setup of possible alleles for 200 loci with $r_{md} = -0.18$. We followed the descriptions of the main article up to the calculation of the preliminary allele effects $\tilde{\mathbf{E}}_1$ and $\tilde{\mathbf{E}}_2$ according to Eq. 3 of the main article. Afterwards, we placed the 200 loci randomly on 12 chromosomes, with chromosome lengths and recombination probabilities as in [41]. Only then did we apply the correction of the additive genetic variance according to Eq. 4 of the main article. With this genetic setup, we performed 20 repetitions of simulations as in setting $\text{FL400}^{300\ \text{BQs}}_{-0.18}$. Table 1 compares the outcomes of this simulation setup with the original results for setting $\text{FL400}^{300\ \text{BQs}}_{-0.18}$.

Table 1. Comparison of simulations

	genetic gain					
	after 20 years			after 100 years		
	mat. eff.	dir. eff.	PC	mat. eff.	dir. eff.	PC
with linkage	3.35	4.82	8.29	10.39	16.84	27.23
without linkage	2.03	4.67	7.73	10.06	15.75	25.83
	genetic variance					
	after 20 years			after 100 years		
	mat. eff.	dir. eff.	PC	mat. eff.	dir. eff.	PC
with linkage	0.799	1.669	1.122	0.249	0.422	0.214
without linkage	0.787	1.537	1.036	0.246	0.427	0.206
	Bias of EBV					
	after 20 years			after 100 years		
	mat. eff.	dir. eff.	SC	mat. eff.	dir. eff.	SC
with linkage	-0.129	-0.021	-0.188	2.594	3.260	5.830
without linkage	0.045	0.094	2.714	4.181	6.904	

Comparisons of Simulations in setting $\text{FL400}^{300\ \text{BQs}}_{-0.18}$, with and without linkage.

We did not find any qualitative differences in the genetic gain and the loss of genetic variance over time. The slower drift rate due to linkage caused a weaker bias in the

estimated breeding values and thus to a more accurate breeding value estimation. This effect can explain the slightly higher total genetic gain in the model with linkage.

References

41. Gupta P, Conrad T, Spötter A, Reinsch N, Bienefeld K. Simulating a base population in honey bee for molecular genetic studies. Genet Sel Evol. 2012;44(14). doi:10.1186/1297-9686-44-14.
