# Supplementary material for: Comparison of infinitesimal and finite locus models for long-term breeding simulations with direct and maternal effects at the example of honeybees
Source: PLoS One. 2019 Mar 6;14(3):e0213270. doi: 10.1371/journal.pone.0213270 (PMC6402681; doi:10.1371/journal.pone.0213270)
Supplement: S3 Appendix — Small-scale study on the selection response to direct and maternal genetic effects dependent on the ratio of additive genetic variances. (PDF) [file pone.0213270.s003.pdf]

Selection strength on direct and maternal effects

Our main simulation studies investigated selection of a trait that was influenced maternally and directly. Hereby, the initial additive genetic variance of the direct effects, ${\sigma_A^d}^2$, was always twice as high as the corresponding value for the maternal effects, σ_A^{m2}. We found that selection always focussed primarily on the direct effects, also when we corrected for the different additive genetic variances. These results were in line with findings in [36, 37], where also a higher direct variance was assumed. In order to investigate the influence of different direct and maternal heritabilities on the selection focus, we conducted a small scale investigation based on the setting INF$^{300\ \mathrm{BQs}}$. We let the initial maternal additive variance vary from 1.0 to 2.0 in steps of 0.1 and adjusted the direct additive variance accordingly, so that

$$\sigma_A^{m2} + {\sigma_A^d}^2 = 3$$

remained constant. With each choice of genetic parameters we conducted 20 simulations over the course of 50 years. Fig 1 shows the ratio of direct vs. maternal genetic gain after 50 years before (blue) and after (red) correction for the different genetic standard deviations. The dotted lines indicate the ratios of genetic standard deviations.

We found that with equal maternal and direct heritabilities, the selection for maternal effects was significantly stronger than selection for direct effects, especially when there was a strong negative correlation between the effects. We see the reason in the fact that the BLUP breeding value estimation worked more accurately for the maternal effect than for the direct effect. For $\sigma_A^{md} = -0.25$, the accuracies of the estimated breeding values in the worker groups were 0.45 for the direct and 0.65 for the maternal effect. In the simulations with $\sigma_A^{md} = -0.75$, the respective values were 0.33 and 0.56. Hence a larger difference in the accuracy of estimated breeding values also yielded a larger difference in selection response. When the genetic variance of the direct effects exceeds one and a half times the genetic variance of the maternal effects (${\sigma_A^d}^2 = 1.8$, $\sigma_A^{m2} = 1.2$), the selection focus shifted to the direct effects. The reason is

that the trait with a higher additive variance has an overproportionally high influence on the outcome of the sum of two traits.

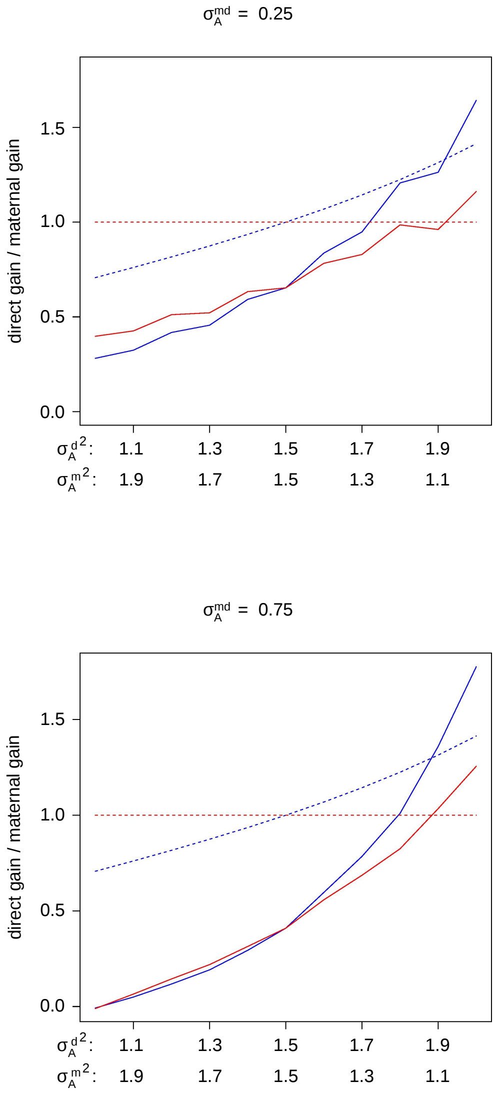

Fig 1. Ratio between direct and maternal genetic gain after 50 years of selection for different rates of initial genetic variance.

References

36. Roehe R, Kennedy BW. The influence of maternal effects on accuracy of evaluation of litter size in swine. J Anim Sci. 1993;71(9):2353–2364. doi:10.2527/1993.7192353x.

37. Roehe R, Kennedy BW. Effect of selection for maternal and direct genetic effects on genetic improvement of litter size in swine. J Anim Sci. 1993;71(11):2891–2904. doi:10.2527/1993.71112891x.
